# Analgesic effect of ropivacaine combined with dexmedetomidine in the postoperative period in children undergoing ultrasound-guided single-shot sacral epidural block: A systematic review and meta-analysis

**DOI:** 10.3389/fped.2023.1099699

**Published:** 2023-03-29

**Authors:** Shoubo Quan, Yuexia Lu, Yujie Huang

**Affiliations:** ^1^ Department of Anesthesiology, Songshan Lake Central Hospital of Dongguan City, Dongguan, China; ^2^ Department of Anesthesiology, Haikou Hospital of Traditional Chinese Medicine, Haikou, China; ^3^ Department of Anesthesiology, Nantong Haimen District People's Hospital, Nantong, China

**Keywords:** dexmedetomidine, ropivacaine, pediatric, sacral epidural block, meta-analysis

## Abstract

**Objective:**

This study aims to evaluate the efficacy of dexmedetomidine as an adjuvant to ropivacaine in prolonging postoperative analgesia and reducing pain scores in children undergoing surgery.

**Methods:**

Five online databases were searched for RCTs on postoperative analgesia of pediatric patients undergoing ultrasound-guided single-shot sacral epidural block with dexmedetomidine as an adjuvant to ropivacaine up to January 2, 2023. Pain score and sedation score at 2, 4, 8, 12, and 24 h after the operation, the time of first receiving additional analgesic drugs, and the number of postoperative adverse effects were selected to compare the efficacy and safety of combined treatment with ropivacaine alone for pediatrics. The standard mean difference (SMD) or odds ratio (OR) and the corresponding 95% confidence interval (95%CI) were calculated by using a random-effects model.

**Results:**

A total of 295 articles were retrieved, but only 20 records were included in this meta-analysis. The results showed that dexmedetomidine combined with ropivacaine for sacral epidural block in children undergoing ultrasound-guided single-shot sacral epidural block had a more prolonged analgesia effect (SMD = 3.47, 95%CI: 2.80, 4.14). There were lower analgesia scores at 2 h(T_1_), 4 h(T_2_), 8 h(T_3_), 12 h(T_4_), and 24 h(T_5_) in postoperative period (**T_1_**: SMD = −1.02, 95%CI: −1.31, −0.72; **T_2_**: SMD = −1.02, 95%CI: −1.32, −0.72; **T_3_**: SMD = −0.84, 95%CI: −1.12, −0.56; **T_4_**: SMD = −0.61, 95%CI: −1.03, −0.20; **T_5_**: SMD = −1.03, 95%CI: −1.28, −0.78). And the incidence of adverse effects was similar between the two groups (OR = 0.84, 95%CI: 0.59, 1.18).

**Conclusions:**

The results of this review and meta-analysis support that dexmedetomidine, as an adjuvant to ropivacaine, can improve postoperative analgesia of surgery and significantly prolong the analgesic time in children, with a similar incidence rate of adverse symptoms when compared with ropivacaine alone.

## Introduction

Due to the particular psychological and physiological characteristics of children, they are prone to severe stress reactions like increased secretion of catecholamines, cortisol, angiotensin II, and cytokines during the perioperative period, the severity of which is closely related to the incidence of postoperative complications. The prevention, evaluation, and treatment of postoperative pain and agitation in children have constantly challenged clinicians and researchers (1–3). The sacral block is the most commonly used pediatric epidural block technique worldwide (4). A meta-analysis showed that sacral block, as a part of multimodal analgesia modality, can lower pain scores, reduce the dosage of general anesthetics, and the occurrence of recovery-related complications, such as nausea, vomiting, motor block, hypoxemia, and analgesic remedies (5). Therefore, the sacral block is often combined with general anesthesia for pediatric surgical anesthesia (5).

It has been reported that about 20% of normal children have anatomical variations such as sacral hiatus malformation or atresia, which increases the incidence of inadequate anesthesia block and local anesthetic toxicity in conventional sacral blockade through blind puncture (6). With the development of ultrasound technology, sacral anesthesia can accurately locate sacral hiatus under the guidance of accurate ultrasound visualization, increase the block success rate, shorten the onset time of the sensory block, and reduce the incidence of repeated punctures and adverse reactions (7). However, local anesthetics are mainly used in the clinical application of sacral block. This single analgesic scheme has a limited duration of analgesia. Although there are dosage forms with a more extended period of action, local anesthetics' analgesic duration is still relatively short (8–10).

Ropivacaine is a long-acting amide local anesthetic commonly used in the practice. It has the characteristics of lower cardiovascular system toxicity and less motor blockade than other long-acting local anesthetics at comparable dosages, such as bupivacaine. It has been widely used in the sacral block in children in recent years. Studies have shown that applying ropivacaine in the ultrasound-guided sacral epidural block can provide a practical analgesic effect for pediatrics (11, 12). However, it still has the disadvantage of a short duration of analgesia.

Dexmedetomidine is a highly selective *α*-2 adrenergic receptor agonist (*α*2: *α*1 = 1620: 1), and its selectivity is eight times that of a similar drug like clonidine. In addition, dexmedetomidine inhibits sympathetic excitability by exciting the *α*2 receptors of the postsynaptic membrane, which reduces the concentration of catecholamines in the blood. So it helps to maintain the heart rate and blood pressure in a relatively constant range and achieves stable hemodynamics and cerebral blood flow (13). Preoperative and perioperative application of dexmedetomidine can relieve the tension of patients, reduce the fear and separation anxiety of children, lower stress response and oxygen consumption, and curtail the dose of other analgesic drugs and related side effects (14, 15). As an adjuvant to local anesthetics, dexmedetomidine accelerates the onset time of local anesthetics and prolongs the action time of local anesthetics [bupivacaine alone: median: 5, 95%CI: 4–6 h; addition of dexmedetomidine: 16, 14–18 h] (16, 17). Presently, relevant studies have shown that adding dexmedetomidine to ropivacaine for an epidural block can extend the analgesic duration of pediatrics without increasing the incidence of side effects, such as nausea, vomiting, and emergence agitation (18).

A timely summary of the anesthesia methods for sacral epidural block in children's lower abdomen will help determine the feasibility and safety of perioperative sedation and analgesia in children in clinical practice. Therefore, this study aimed to conduct a systematic review of published randomized controlled trials (RCTs) comparing the safety and efficacy of dexmedetomidine plus ropivacaine for ultrasound-guided single-shot sacral epidural block in perioperative sedation and analgesia in pediatrics to provide ample quantitative evidence for the use of perioperative anesthesia in pediatric.

## Methods

### Retrieval strategy

According to the inclusion criteria, we searched databases such as WanFang Data, China National Knowledge Infrastructure, PubMed, Web of Science, and Cochrane Library for RCT studies on perioperative analgesia of dexmedetomidine combined with ropivacaine for ultrasound-guided single-shot sacral epidural block in pediatric. The search period was from establishing the database to January 2, 2023. The languages were limited to Chinese and English. The search keywords included: “dexmedetomidine”, “pediatric”, “ropivacaine”, “sacral epidural block”, “caudal block,” “caudal anesthesia”, and “caudal epidural block”. Meanwhile, references of the included articles were manually searched to supplement possible missing literature.

### Inclusion and exclusion criteria

The publication included for meta-analysis should meet the following inclusion criteria: (1) the participants of included paper were pediatric patients undergoing the ultrasound-guided single-shot sacral epidural block; (2) the study design was a randomized controlled study; (3) the study aim was to compare the efficacy and/ or safety of dexmedetomidine combined with ropivacaine and ropivacaine alone for ultrasound-guided single-shot sacral epidural block in pediatric; (4) the primary and secondary outcomes of included publications includes but not limits to the pain score of pediatric patients at 2, 4, 8, 12, 24 h after the operation, the sedation score of pediatric patients at 2, 4, 8, 12, 24 h after the operation, the time of first receiving additional analgesic drugs after the operation and the number of postoperative adverse effects. Publication that met any criteria listed below was excluded from the meta-analysis: (1) publication like case reports, research reviews, conference papers, and master's and doctoral dissertations which couldn't provide available information for meta-analysis was excluded; (2) Articles where the full text of the research cannot be obtained; (3) the data of research is incomplete; (4) the original data cannot be obtained and/ or the data cannot be transformed *via* statistical methods.

### Literature screening

First, two researchers independently searched the Chinese and English databases according to the preset search keywords and eliminated the retrieved duplicate documents using the literature management software (Endnote 20). Second, the two researchers downloaded and read the complete text and selected the included literature according to the inclusion and exclusion criteria. Next, the two researchers compared the results and consulted a third researcher to arbitrate if there was a disagreement. If the experimental research data was incomplete, the author should be contacted by email to obtain the original experimental result data, and the article was excluded if the experimental research data could not be collected. For papers whose original data cannot be obtained intuitively, if there was no result in contacting the author, data conversion could be performed through the existing charts and graphs. The article would be excluded if the error was huge and the conversion failed.

### Data extraction and quality evaluation

Two researchers extracted the data from the final included literature, then checked and organized them into a table. The data to be removed were as follows: (1) Basic information of the included studies: author name, report date, sample size, specific methods of nerve block, and dose and concentration of ropivacaine and dexmedetomidine, (2) Main outcome indicators: pain score and sedation score at 2, 4, 8, 12, 24 h after the operation, the time of first receiving additional analgesic drugs and the number of postoperative adverse effects. Pain scores included the visual analogue scale (VAS), Face, Legs, Activity, Cry and Consolability scale (FLACC), and Children's Hospital of Eastern Ontario Pain Scale (CHEOPS). Ramsay sedation score was used for the sedation scale. If graphs and charts analyze the outcome indicators in the literature, the author should be contacted to obtain the original data. If it was unavailable, the two researchers used the data extraction software GetData Graph Digitizer to extract effective outcome indicators. The existing conversion calculation method was able to convert the experimental study using the interquartile range to analyze the outcome index data.

Two researchers evaluated the methodological quality of all included studies according to the bias risk assessment tool in the Cochrane Handbook. The main evaluation criteria included random sequence generation, allocation concealment, blinding (subjects and experimenters), blinding of research outcomes, data integrity of outcome indicators, selective reporting, and other biases. The evaluation criteria used three ratings, “unclear,” “high-risk bias,” and “low-risk bias,” to determine the study's quality.

### Statistical methods

STATA version 15.1 software was used to analyze the included literature data statistically (19, 20). In consideration of clinical heterogeneities in the subjects undergoing different surgery, differences in age between the subjects and the evaluation methods used for pain scoring, this meta-analysis used a random-effects model to combine the effect size. In addition, this research conducted a subgroup analysis according to various studies' different pain scoring methods. It aimed to compare the analgesic effects and safety of ropivacaine combined with dexmedetomidine and ropivacaine alone at different doses and evaluation methods. We used the standardized mean difference (SMD) and its 95% confidence interval (95%CI) for continuous variables to evaluate the meta-analysis results. The odds ratio (OR) with 95% CI was used for dichotomous variables. In addition, the *χ*^2^ test was performed on the included studies. The heterogeneity was determined by combining *I^2^* and *P* values. If *I^2^* ≤ 0.5, *P* ≥ 0.1, there was no heterogeneity between studies; if *I^2^* > 0.5, *P* < 0.1, there was heterogeneity. We used forest plots to show the results of the meta-analysis. Additionally, we assessed the publication bias of the results using Egger's test and funnel plot. The sensitivity of the results was evaluated by Duval and Tweedie's trim and fill test (21, 22). We would provide exact *P* values unless *P* < 0.01. *P* < 0.05 was considered statistically significant, except for *P* < 0.10 in Egger's test, which was considered statistically significant.

## Results

### Literature retrieval results and basic characteristics of included studies

**Figure 1:**
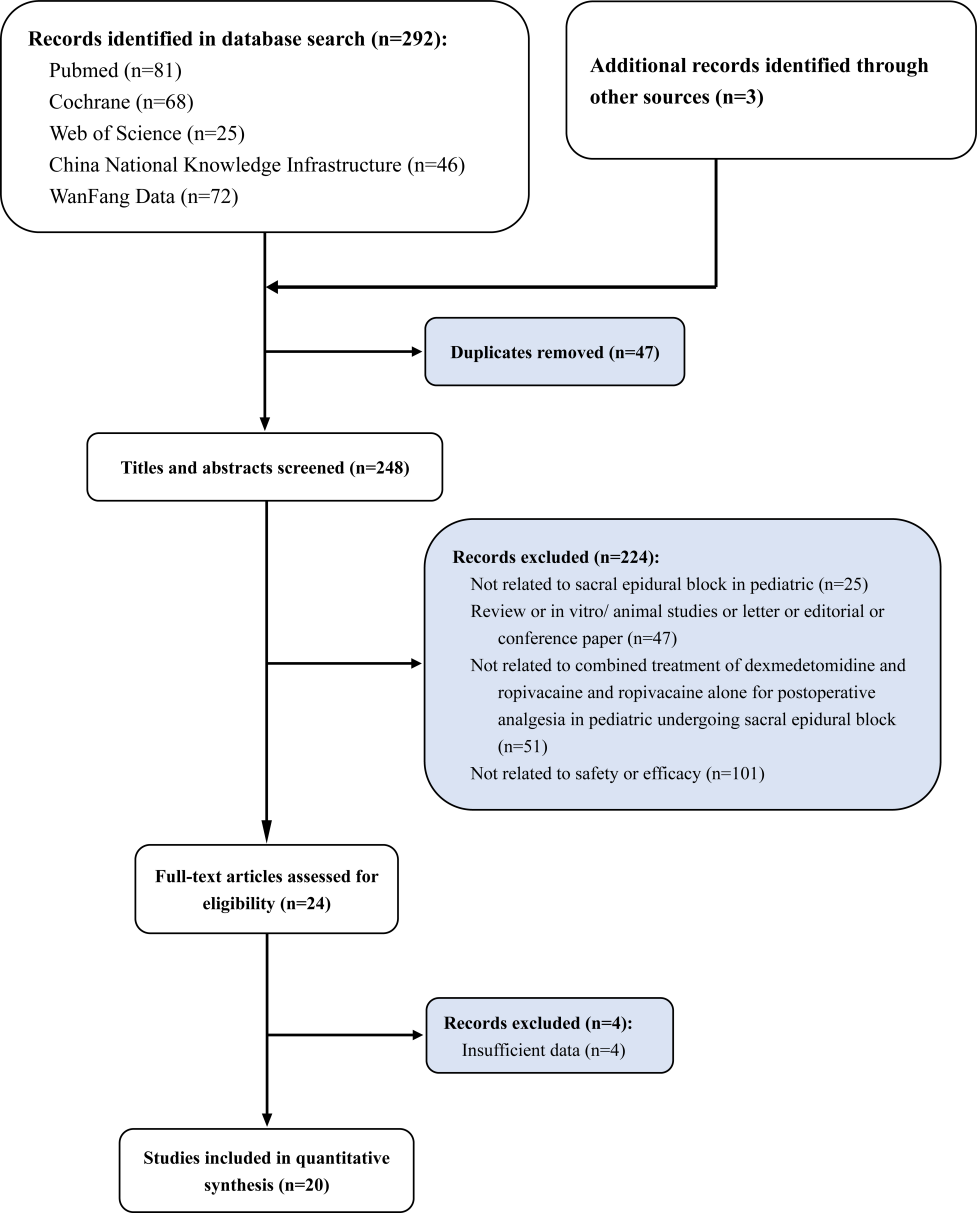
Study selection flowchart, systematic review and meta-analysis of analgesic effect of ropivacaine combined with dexmedetomidine in postoperative period in children undergoing ultrasound-guided single-shot sacral epidural block.

A total of 295 articles were initially retrieved according to our search strategy. And 47 repeated articles were identified and deleted by the software Endnote 20. Two independent researchers screened the retrieved articles according to the following items. Whether the literature was related to sacral epidural block in pediatric (*n* = 25). Whether the article was a meeting summary, review, etc., with no effective outcome indicators or available data (*n* = 47). Whether the article compared dexmedetomidine combined with ropivacaine and ropivacaine alone for postoperative analgesia in pediatrics undergoing sacral epidural block (*n* = 51). Whether the article provided evaluation indicators of the effectiveness and safety of the two anesthesia methods (*n* = 101). The screening flow chart is shown in Figure 1. Finally, we only included 20 RCT studies for quantitative analysis. A total of 830 children with combined anesthesia and 672 children with ropivacaine alone in local anesthesia were included in this meta-analysis. The essential characteristics are shown in Table 1 (23–42).

**Table 1:** Baseline characteristics of included studies for meta-analysis.

First author, year	No. of cases		Age (year, Comb/Ctrl)	Surgery	Dose/administration of Combined group and control group
	Comb	Ctrl			
Zhao Y, 2019 (23)	40	40	3.35 ± 1.29/3.20 ± 1.24	Concealed penis lengthening surgery	Combined group: 1 ug/kg dexmedetomidine + 0.15% ropivacaine. Control group: normal saline + 0.15% ropivacaine.
Wu QL, 2021 (24)	45	45	2.59 ± 0.94/2.73 ± 1.12	Laparoscopic high ligation of hernia sac	Combined group: 1 ug/kg dexmedetomidine + 0.25% ropivacaine. Control group: normal saline + 0.25% ropivacaine.
Lin *P*, 2020 (25)	46	46	2.67 ± 1.05/2.80 ± 1.12	Correction of hypospadias	Combined group: 0.7 ug/kg dexmedetomidine + 0.2% ropivacaine. Control group: normal saline + 0.2% ropivacaine.
Wang CG, 2018 (26)	40	20	4.40 ± 1.35/4.60 ± 1.00	High ligation of hernia sac	Combined group A: 2.0 ug/kg dexmedetomidine + 0.25% ropivacaine; Combined group B: 1.0 ug/kg dexmedetomidine + 0.25% ropivacaine. Control group: normal saline + 0.25% ropivacaine.
Zhu F, 2019 (27)	23	23	3.21 ± 0.99/4.10 ± 1.32	Children hip development dysplasia correction surgery	Combined group: 1.0 ug/kg dexmedetomidine + 0.2% ropivacaine. Control group: normal saline + 0.2% ropivacaine.
Sun WG, 2021 (28)	45	45	2.32 ± 0.87/2.64 ± 0.91	Correction of hypospadias	Combined group: 1.0 ug/kg dexmedetomidine + 0.15% ropivacaine. Control group: normal saline + 0.15% ropivacaine.
Zeng Y, 2020 (29)	27	27	3.70 ± 1.03/3.90 ± 0.91	Correction of hypospadias	Combined group: 1.0 ug/kg dexmedetomidine + 0.15% ropivacaine. Control group: normal saline + 0.15% ropivacaine.
Zhou Y, 2014 (30)	32	31	3.01 ± 1.32/3.32 ± 1.18	Unilateral inguinal hernia surgery	Combined group: 1 ug/kg dexmedetomidine + 0.25% ropivacaine. Control group: normal saline + 0.25% ropivacaine.
Yuan YQ, 2017 (31)	30	15	4.00 ± 1.20/3.80 ± 1.40	Unilateral inguinal hernia surgery	Combined group A: 2.0 ug/kg dexmedetomidine + 0.25% ropivacaine; Combined group B: 1.0 ug/kg dexmedetomidine + 0.25% ropivacaine. Control group: normal saline + 0.25% ropivacaine.
Liu SM, 2017 (32)	30	30	3.53 ± 1.28/3.20 ± 1.19	Congenital dislocation hip surgery	Combined group: 1.0 ug/kg dexmedetomidine + 0.2% ropivacaine. Control group: normal saline + 0.2% ropivacaine.
Yang SH, 2017 (33)	40	40	3.53 ± 1.28/3.20 ± 1.19	Correction of hypospadias	Combined group: 2.0 ug/kg dexmedetomidine + 0.15% ropivacaine. Control group: normal saline + 0.15% ropivacaine.
Liu JF, 2014 (34)	40	20	3.60 ± 1.40/3.30 ± 1.20	Inguinal hernia surgery	Combined group A: 2.0 ug/kg dexmedetomidine + 0.25% ropivacaine; Combined group B: 1.0 ug/kg dexmedetomidine + 0.25% ropivacaine. Control group: normal saline + 0.25% ropivacaine.
Wu QL, 2021 (35)	45	45	2.70 ± 1.10/2.60 ± 1.30	High ligation of hernia sac	Combined group: 1 ug/kg dexmedetomidine + 0.25% ropivacaine. Control group: normal saline + 0.25% ropivacaine.
Wang Y, 2020 (36)	80	80	4.40 ± 1.40/4.60 ± 1.30	Testicular descent and fixation, penile lengthening and Hypospadioplasty	Combined group: 1 ug/kg dexmedetomidine + 0.25% ropivacaine. Control group: normal saline + 0.25% ropivacaine.
Liao YC, 2019 (37)	44	22	3.30 ± 1.60/3.20 ± 1.70	Laparoscopic repair of oblique inguinal hernia and high ligation of sheathoid process	Combined group A: 2.0 ug/kg dexmedetomidine + 0.15% ropivacaine; Combined group B: 1.0 ug/kg dexmedetomidine + 0.15% ropivacaine. Control group: normal saline + 0.25% ropivacaine.
Wang Z, 2015 (38)	20	20	5.37 ± 1.66/5.52 ± 1.54	Correction of hypospadias	Combined group: 2.0 ug/kg dexmedetomidine + 0.25% ropivacaine. Control group: normal saline + 0.25% ropivacaine.
Wang Y, 2021 (39)	120	40	4.30 ± 1.30/4.30 ± 1.30	Correction of hypospadias	Combined group A: 2.0 ug/kg dexmedetomidine + 0.25% ropivacaine; Combined group B: 1.5 ug/kg dexmedetomidine + 0.25% ropivacaine; Combined group C: 1.0 ug/kg dexmedetomidine + 0.25% ropivacaine. Control group: normal saline + 0.25% ropivacaine.
Imani, F, 2021 (40)	23	23	4.80 ± 1.10/4.50 ± 1.20	Lower abdominal surgery	Combined group: 2.0 ug/kg dexmedetomidine + 0.2% ropivacaine. Control group: normal saline + 0.2% ropivacaine.
Kamal, M, 2016 (41)	30	30	4.23 ± 2.43/3.93 ± 2.06	Lower abdominal surgery	Combined group: 2.0 ug/kg dexmedetomidine + 0.25% ropivacaine. Control group: normal saline + 0.25% ropivacaine.
Sarvesh, B, 2019 (42)	30	30	6.50 ± 2.92/6.67 ± 2.83	Infra-umbilical surgery	Combined group: 1 ug/kg dexmedetomidine + 0.25% ropivacaine. Control group: normal saline + 0.25% ropivacaine.

Comb, combined treatment of ropivacaine and dexmedetomidine; Ctrl, control; y, year.

**Figure 2:**
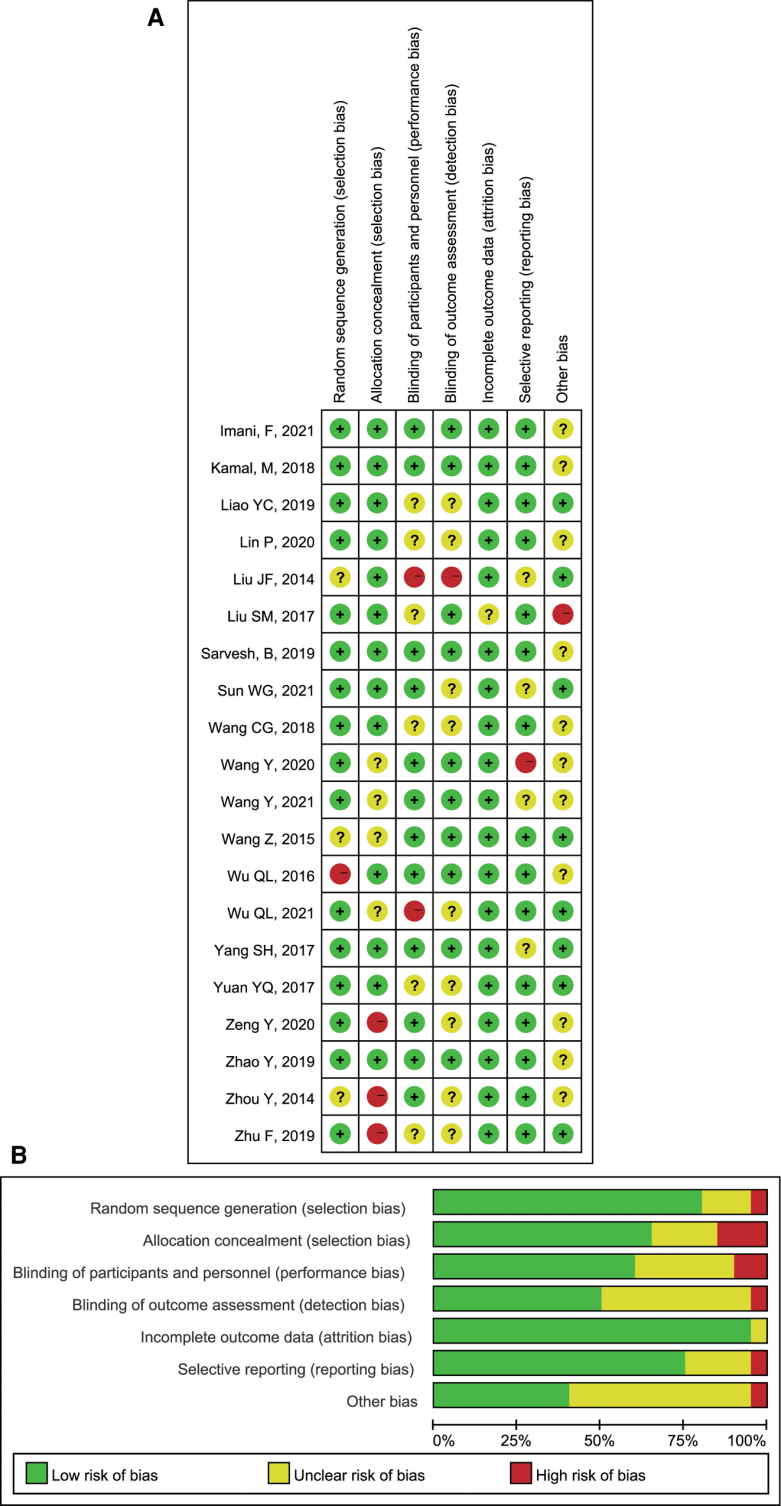
Review authors’ judgements: (**A**) Risk of bias summary; (**B**) Risk of bias graph presented as percentages.

Two researchers assessed the risk of bias in the included studies according to the bias risk assessment tool in the Cochrane Handbook. In the included trials, ten pieces of literature were randomly grouped *via* computer; six papers were grouped by random number table; thirteen essays were assigned and concealed by sealed envelopes; three papers were not assigned and hidden; nine papers were double-blind studies (patients and evaluators were blinded). All test outcome indicators were complete. A detailed risk of bias assessment chart for the literature is as follows: risk of bias map for included studies (Figure 2A); a summary of the risk of bias of included studies (Figure 2B).

## Meta-analysis results

### Comparison of pain scores at 2 h, 4 h, 8 h, 12 h, and 24 h after operation

**Figure 3:**
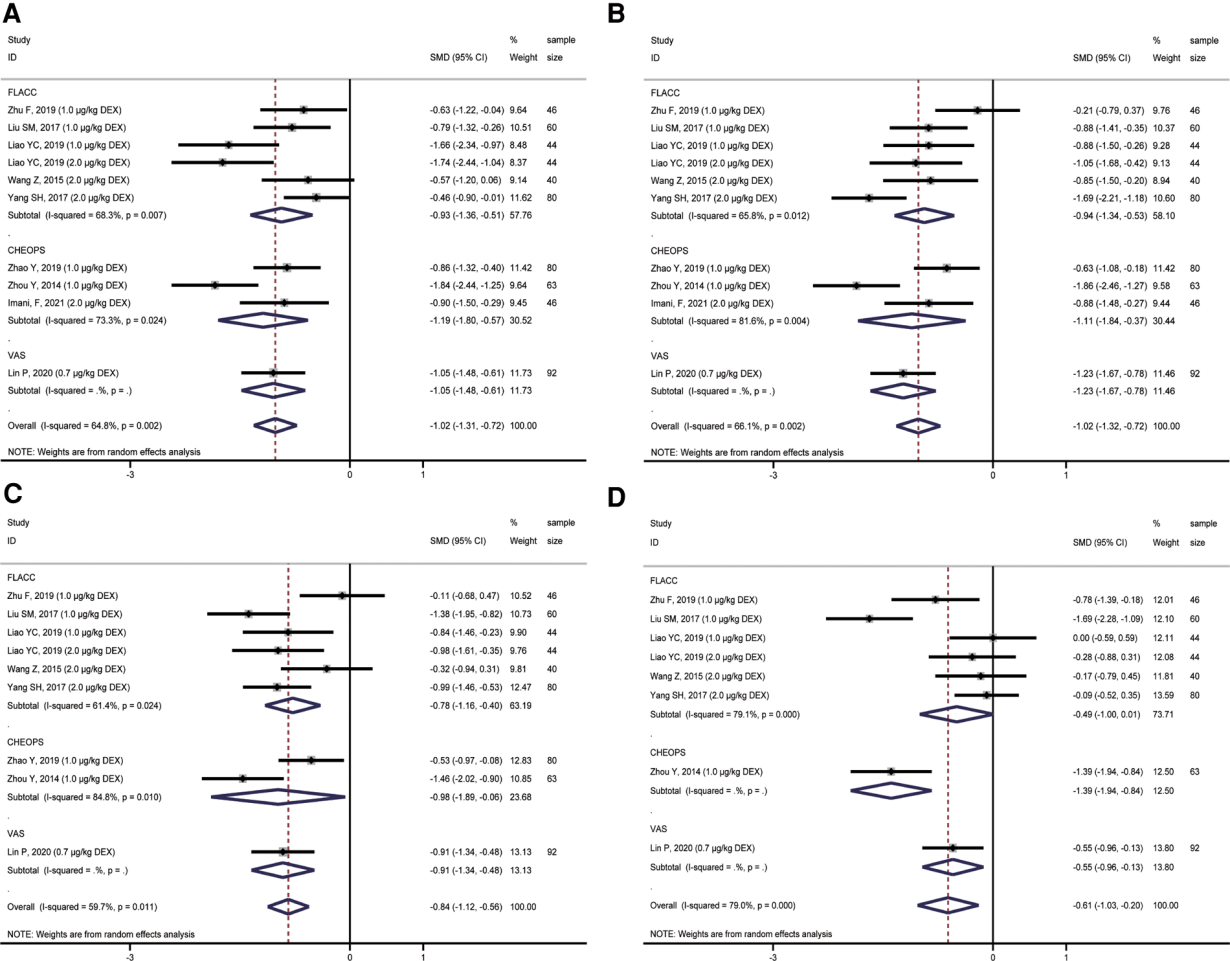
Forest plot of comparison of combined group and control group: (**A**) Pain score at 2 h (T_1_); (**B**) Pain score at 4 h (T_2_); (**C**) Pain score at 8 h (T_3_); (**D**) Pain score at 12 h (T_4_).

**Figure 4:**
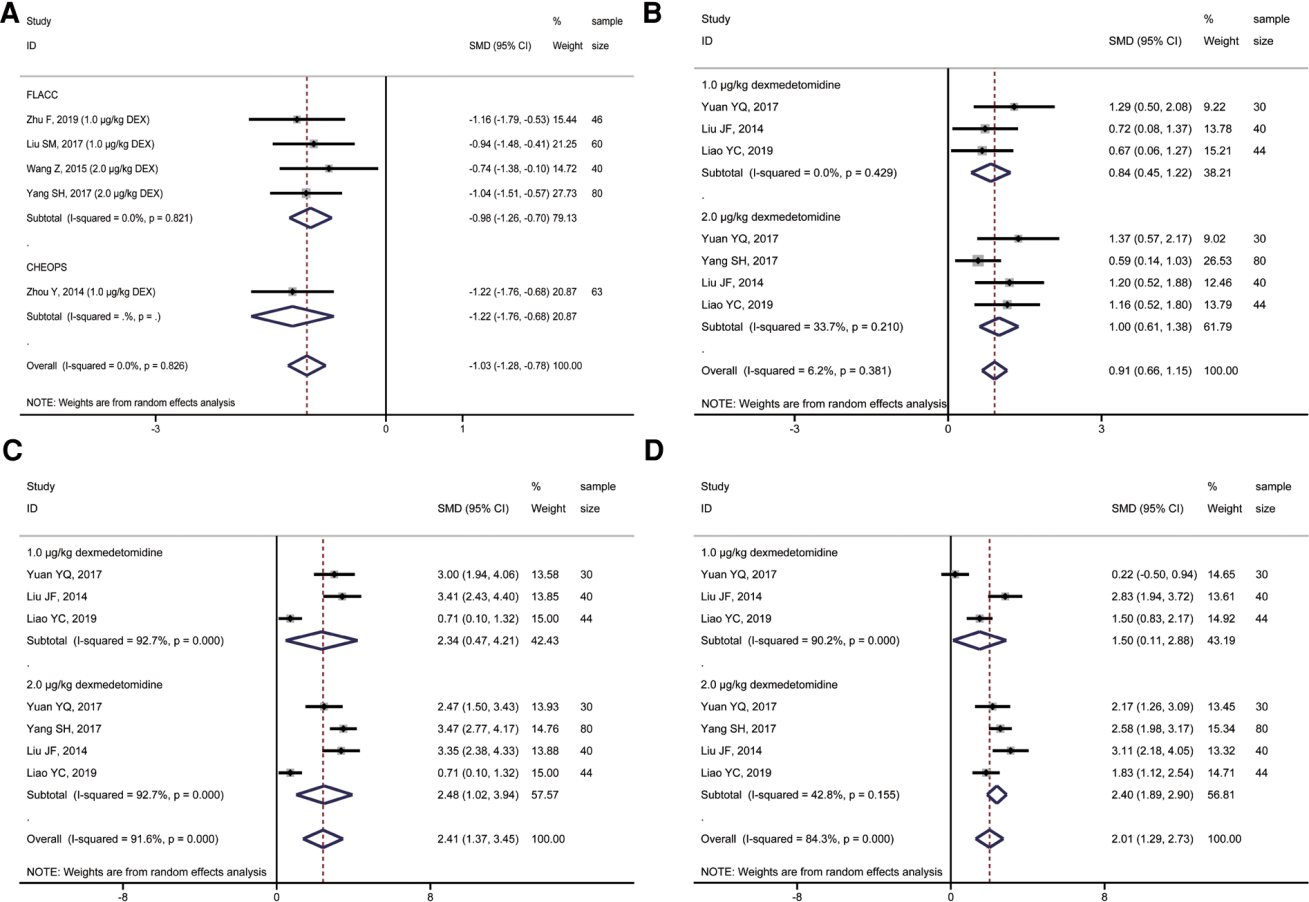
Forest plot of comparison of combined group and control group: (**A**) Pain score at 24 h (T_5_); (**B**) Ramsay score at 2 h (T_1_); (**C**) Ramsay score at 4 h (T_2_); (**D**) Ramsay score at 8 h (T_3_).

Nine studies were included in the quantitative analysis, involving 551 patients reporting postoperative pain scores. Due to the different ages of children included in each study, three scoring methods of FLACC, CHEOPS, and VAS were used to score children's postoperative pain. We performed a subgroup analysis of postoperative pain in children with different scoring methods. The results showed that the analgesic effects of ropivacaine combined with dexmedetomidine in undergoing sacral anesthesia were better than those of ropivacaine alone at 2 h (T_1_), 4 h (T_2_), 8 h (T_3_), 12 h (T_4_) and 24 h (T_5_) after surgery (**T_1_**: SMD = −1.02, 95%CI: −1.31, −0.72; **T_2_**: SMD = −1.02, 95%CI: −1.32, −0.72; **T_3_**: SMD = −0.84, 95%CI: −1.12, −0.56; **T_4_**: SMD = −0.61, 95%CI: −1.03, −0.20; **T_5_**: SMD = −1.03, 95%CI: −1.28, −0.78; Figures 3A–D, 4A). However, there was unneglected heterogeneity among the studies (**T_1_**: *I^2 ^*= 64.8%, *P* = 0.002; **T_2_**: *I^2 ^*= 66.1%, *P* = 0.002; **T_3_**: *I^2 ^*= 59.7%, *P* = 0.011; **T_4_**: *I^2 ^*= 79.0%, *P* < 0.001; **T_5_**: *I^2 ^*= 0.0%, *P* = 0.826).

### Comparison of sedation scores at 2 h, 4 h, 8 h, 12 h, and 24 h after operation

**Figure 5:**
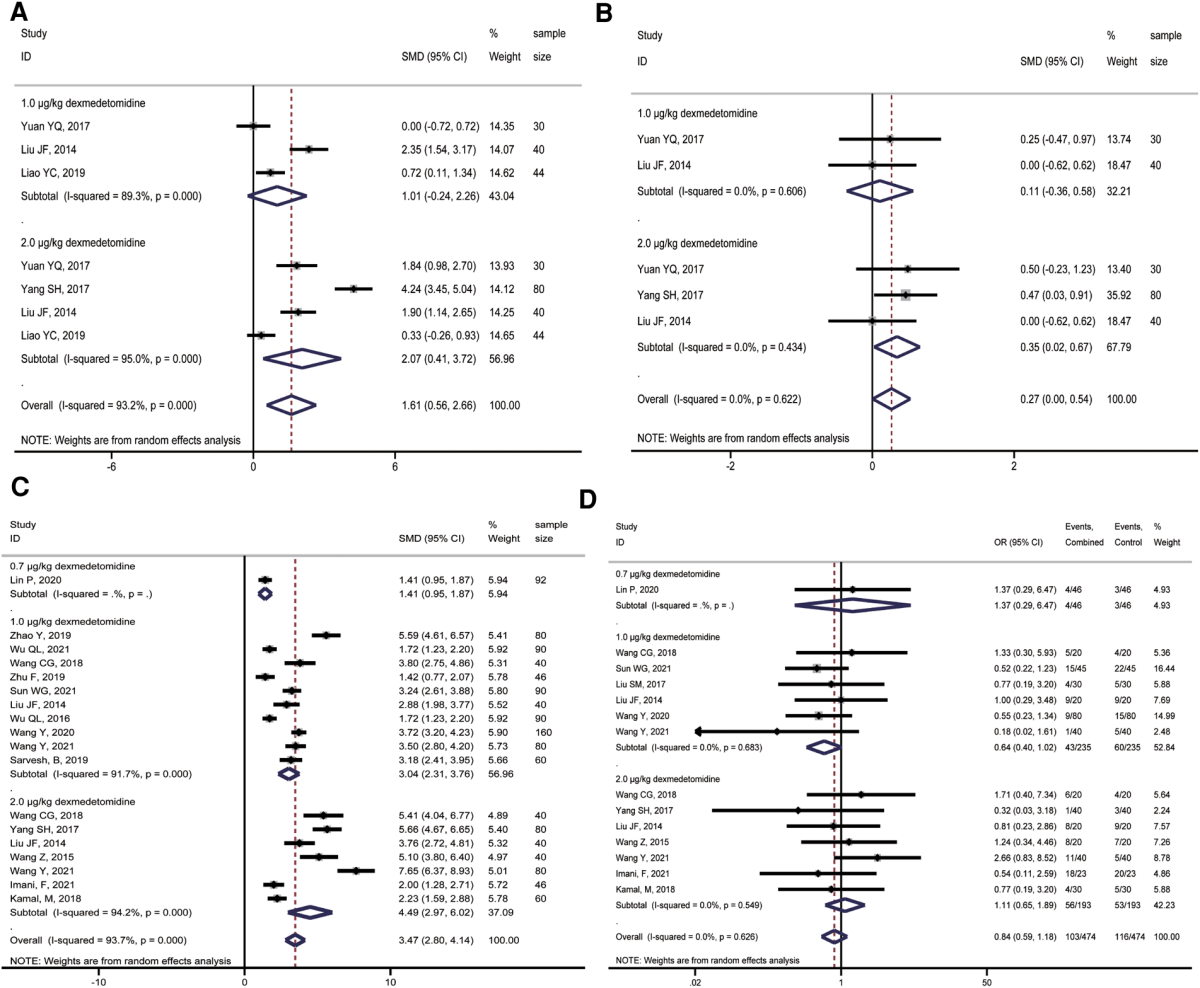
Forest plot of comparison of combined group and control group: (**A**) Ramsay score at 12 h (T_4_); (**B**) Ramsay score at 24 h (T5); (**C**) First postoperative analgesic supplementation time; (**D**) Incidence of postoperative adverse effects.

A total of 4 studies compared postoperative sedation scores, including 194 children. All four studies used the Ramsay score to evaluate the sedative effect on children after the operation. However, the impact of different doses of ropivacaine combined with dexmedetomidine on sacral anesthesia in children was compared in various studies. A subgroup analysis of different doses of dexmedetomidine was performed to observe the possible adverse effects of high doses of dexmedetomidine on children. The results showed that, compared with ropivacaine alone, the application of ropivacaine combined with dexmedetomidine in sacral anesthesia after 2 h (T_1_), 4 h (T_2_), and 8 h (T_3_) could achieve an excellent sedative effect (2,3 Ramsay score) (**T_1_**: SMD = 0.91, 95%CI: 0.66, 1.15; **T_2_**: SMD = 2.41, 95%CI: 1.37, 3.45; **T_3_**: SMD = 2.01, 95%CI: 1.29, 2.73; Figures 4B–D). However, only 2 *μ*g/kg dexmedetomidine combined with ropivacaine in sacral anesthesia still had a better sedative effect than ropivacaine alone at T_4_. The sedative effect of the remaining concentrations of dexmedetomidine combined with ropivacaine was not statistically significant at T_4_ and time after this moment when compared with ropivacaine alone (Figures 5A,B).

### Comparison of the first postoperative analgesic supplementation time

A total of 15 studies reported the time of first use of analgesic drugs after surgery. And a random-effects model was applied to calculate the combined effect. The first time of receiving analgesic drugs in the combined group with 0.7 μg/kg, 1.0 μg/kg, and 2.0 μg/kg dexmedetomidine were longer than those of ropivacaine alone, with a dose-response relationship, and the differences were statistically significant (**0.7** **μg/kg**: SMD = 1.41, 95%CI: 0.95, 1.87; **1.0** **μg/kg**: SMD = 3.04, 95%CI: 2.31, 3.76; **2.0 μg/kg**: SMD = 4.49, 95%CI: 2.97, 6.02; Figure 5C). However, there was significant heterogeneity among studies (**1.0** **μg/kg**: *I^2 ^*= 91.7%, *P* < 0.001; **2.0** **μg/kg**: *I^2 ^*= 94.2%, *P* < 0.001).

### Comparison of incidence of postoperative effects reactions

Eleven studies compared the incidence of adverse effects between the combined group and ropivacaine alone. The meta-analysis showed that the incidence of adverse effects in the combined group was lower than in the ropivacaine-only group. However, the difference was not statistically significant (OR = 0.84, 95%CI: 0.59, 1.18; Figure 5D).

### Publication bias detection and sensitivity analysis

We conducted a publication bias test on the indicators of postoperative pain score, postoperative sedation score, postoperative first analgesic drug supplementation time, and postoperative adverse effect rate. The results showed no significant publication bias in the above four indicators (Table 2). Besides, the funnel plot showed the same results of Egger's test (Supplementary Figures S1A–D, S2A–C). In addition, the sensitivity analysis results of the four indicators suggested that the effect sizes of the meta-analysis results were stable. Moreover, there was no essential change before and after the indicators were trimmed and filled, with clear guiding significance (Table 2).

**Table 2:** Evaluation of publication bias and sensitivity analysis.

Index	Egger's regression		Duval and Tweedie's trim and fill		
	Intercept	*p*	Original effect size	Studies trimmed	Adjusted effect size
**Analgesic effect score**
T_1_	−4.835	0.133	−1.02 (−1.31, −0.72)	0	−1.02 (−1.31, −0.72)
T_2_	0.501	0.910	−1.02 (−1.32, −0.72)	2	−1.18 (−1.51, −0.85)
T_3_	0.371	0.927	−0.84 (−1.12, −0.56)	0	−0.84 (−1.12, −0.56)
T_4_	−3.110	0.578	−0.61 (−1.03, −0.20)	0	−0.61 (−1.03, −0.20)
T_5_	1.298	0.654	−1.03 (−1.28, −0.78)	0	−1.03 (−1.28, −0.78)
**Ramsay score**
T_1_	4.300	0.114	0.91 (0.66, 1.15)	2	0.82 (0.57, 1.07)
T_2_	5.255	0.164	2.41 (1.37, 3.46)	3	1.42 (0.29, 2.55)
T_3_	4.005	0.553	2.01 (1.29, 2.74)	2	1.68 (0.98, 0.37)
T_4_	9.236	0.187	1.61 (0.56, 2.66)	0	1.61 (0.56, 2.66)
T_5_	−1.473	0.522	0.27 (0.00, 0.54)	0	0.27 (0.00, 0.54)
Analgesic duration	5.208	0.102	3.47 (2.80, 4.14)	6	2.46 (1.73, 3.19)
Adverse events	0.255	0.816	0.99 (0.65, 1.34)	0	0.99 (0.65, 1.34)

T_1_, measuring at 2 h after operation; T_2_, measuring at 4 h after operation; T_3_, measuring at 8 h after operation; T_4_, measuring at 12 h after operation; T_5_, measuring at 24 h after operation.

## Discussion

### Summary of results

Our study revealed that combined treatment of ropivacaine and dexmedetomidine presented a lower pain score than ropivacaine alone treatment in pediatrics undergoing ultrasound-guided single-shot sacral epidural block. It was consistent in subgroup analysis on different pain scoring methods. Moreover, the application of combined treatment in sacral anesthesia could achieve an excellent sedative effect when compared with ropivacaine alone treatment. The first time of receiving analgesic drugs in the combined group with 0.7 μg/kg, 1.0 μg/kg, and 2.0 μg/kg dexmedetomidine were longer than those of ropivacaine alone, with a dose-response relationship. The incidences of adverse effects were similar in the combined group and the ropivacaine alone group.

In 1980, Cork first described the ultrasound imaging of adult epidural space. Ultrasound imaging plays a vital role in confirming the site of sacral canal injection and the visualization process, thus affecting the success rate of the block (43). It is reported that a typical blind sacrococcygeal epidural's success rate is about 75% in pediatric patients, and ultrasound makes up for the 25% failure rate (44). Ultrasound can more accurately determine the position of the puncture needle than the Swoosh test which is a simple and accurate test to confirm successful caudal insertion in children with a 95.6% success rate of caudal anaesthesia (45). The implementation of ultrasound-guided anesthesia technology improves the anesthesia quality of regional anesthesia, with a highly successful block rate, less local anesthetic dose, fast onset speed, increased patient satisfaction, and exemplary safety (7, 46). Notably, the immobilization of local anesthetics in infants is reduced, and diffusion is increased. Due to the increase of epidural fat fluidity and the decrease of aggregated fat, the onset time of local anesthetics shortens, and the longitudinal diffusion and surrounding diffusion of local drugs extend. In addition, the secondary release of local anesthetics is reduced, and the duration of action is diminished (47). Applying local anesthetic adjuvant is one of the methods to improve the pain rebound after anesthesia regression. The research on dexmedetomidine as a local anesthetic adjuvant is increasing to reduce the toxic and side effects of local anesthetic adjuvants on children. Therefore, this meta-analysis aimed to summarize the published studies on the perioperative analgesic effect and prolonged analgesic time of ultrasound-guided single-shot sacral epidural block with dexmedetomidine added to ropivacaine in pediatrics.

The analgesic effect of dexmedetomidine as an adjuvant for sacral epidural block anesthesia in children within 24 h after surgery was notable. The meta-analysis results showed that the pooled effect values of studies using FLACC, CHEOPS, and VAS evaluation tools received lower pain scores from 2 h to 24 h after surgery, and the difference was statistically significant. However, there was considerable heterogeneity among studies, which may be related to different evaluation tools, types of surgery, and doses of dexmedetomidine. The forest plot indicated that none study showed higher pain scores in the combined group than in the control group, which essentially affirmed the combined group sacral epidural block anesthesia effect. In addition, we did not observe the results that affected the current conclusions from publication bias detection and sensitivity analysis. In addition to the studies that can be converted into quantitative analysis, the results of two studies that cannot convert the articles ‘ charts into quantitative analysis data also concluded consistent with this meta-analysis. Adding dexmedetomidine could effectively improve the analgesic effect of ropivacaine sacral epidural block, and the postoperative pain score of children is lower (18, 48).

Sufficient sedation (2, 3 Ramsay score) was essential in pediatrics after surgery. As the dose of dexmedetomidine increased, the Ramsay sedation score in the combination group was greater than that in the control group but did not exceed the grade 3 Ramsay score. The maximum concentration of dexmedetomidine used in the study included in the systematic review was 2.0 μg/kg. The results were that the higher the concentration, the longer the duration of adequate sedation in the children, but the addition of dexmedetomidine at any concentration at 24 h was no different from the control group. However, only three studies evaluated the sedative effects of 24 h in children. Additional studies are still needed to explore the long-term sedative effects of dexmedetomidine combined with ropivacaine sacral epidural block in children. In addition to papers included for quantitative analysis, other six publications reported that combined treatment presented adequate sedative effect in pediatrics undergoing-ultrasound guided single-shot sacral epidural block, with statistically significant. These papers were not included for quantitative analysis because their data in full-text can't provide or transform into data available for meta-analysis. It corroborated our quantitative results again (26, 28, 32, 35, 36, 38).

Dexmedetomidine combined with ropivacaine used in sacral epidural block can effectively extend the duration of local anesthetic analgesia. This meta-analysis showed that 0.7 μg/kg, 1.0 μg/kg, and 2.0 μg/kg dexmedetomidine could effectively prolong the duration of analgesia in ropivacaine sacral epidural anesthesia, with a dose-response relationship. There was no obvious publication bias, and the sensitivity analysis results were stable. Although the difference between the surgery and the patient's baseline characteristics may lead to the high heterogeneity of the research results, each study in the forest plot indicated that dexmedetomidine could effectively prolong the analgesic duration of ropivacaine sacral epidural block. In addition, it was worth noting that adding dexmedetomidine as a local anesthetic adjuvant reduced the incidence of postoperative adverse effects in the combined group, but the difference was not statistically significant compared with the control group. It might be related to dexmedetomidine promoting hemodynamic stability.

This meta-analysis study showed that dexmedetomidine combined with ropivacaine sacral epidural block could significantly improve analgesia and sedation, substantially prolong the analgesia time of sacral anesthesia, and reduce the incidence of adverse effects in children. Its mechanism of action was as follows. First, dexmedetomidine can enter the sacral canal and directly activate the *α*2 receptor in the spinal dorsal horn to produce an analgesic effect. Next, dexmedetomidine was absorbed into the blood through the sacral canal capillaries, activating the central nervous system and peripheral nerve endings' adrenaline *α*2 receptor. As a result, cells' hyperpolarization reduces pain signals to the brain conduction and inhibits substance *P* and other harmful peptides, resulting in an analgesic effect (49–51). In addition, the inhibition of the sympathetic nerve and enhancement of the vagus nerve of dexmedetomidine could significantly reduce the use of perioperative anesthetics which effectively lower respiratory inhibition, relieve stress response, promote hemodynamic stability and reduce postoperative pain response to improve perioperative comfort of patients (52). Therefore, dexmedetomidine is an ideal adjuvant for local analgesic drugs for children and neonates.

Limitations: The main limitations of this meta-analysis are as follows. First, there was a high heterogeneity among the studies of this meta-analysis, which might overestimate the perioperative analgesic effect of dexmedetomidine as an adjuvant for sacral epidural anesthesia in children. However, we believed that it might be associated with different lower abdominal surgeries between various studies, varied doses of dexmedetomidine and ropivacaine, and differences in the demographic characteristics of children. Second, there were few studies on the sedative effect of dexmedetomidine combined with ropivacaine for the sacral epidural block on children during the perioperative period. In the future, more rigorous studies with large sample sizes are needed to supplement the results of this meta-analysis.

## Conclusion

In summary, this meta-analysis supports that dexmedetomidine, an adjuvant to ropivacaine, can improve postoperative analgesia in pediatrics undergoing ultrasound-guided single-shot sacral epidural block. It can significantly prolong the analgesic time of sacral anesthesia. Also, the incidence of adverse effects of dexmedetomidine combined with ropivacaine was similar to that of ropivacaine alone.

## Data Availability

The raw data supporting the conclusions of this article will be made available by the authors, without undue reservation.

## Supplementary material

Supplementary Figure 1 Funnel plot of comparison of combined group and control group: A. Pain score at 2h (T1); B. Pain score at 4h (T2); C. Pain score at 8h (T3); D. Pain score at 12h (T4).

Supplementary Figure 2 Funnel plot of comparison of combined group and control group: A. Pain score at 24h (T5); B. First postoperative analgesic supplementation time; C. Incidence of postoperative adverse effects.
